# Evaluation of Serum Adipokines in Peripheral Arterial Occlusive Disease

**DOI:** 10.1155/2012/257808

**Published:** 2012-04-04

**Authors:** Claudia D. Gherman, Aurel I. Mironiuc

**Affiliations:** Surgical Clinic II, “Iuliu Hatieganu” University of Medicine and Pharmacy Cluj-Napoca, No. 4-6 Clinicilor Street, 400006 Cluj-Napoca, Romania

## Abstract

*Aim*. Out study aimed to assess the serum levels of adipokines in patients with peripheral arterial occlusive disease (PAOD) caused by atherosclerosis. *Methods*. Serum samples were obtained from 221 patients. One hundred and forty patients, (26 females and 114 males) met the inclusion criteria and were assigned into the case group. Eighty one patients (17 females and 64 males), were included in the control group. Circulating plasma levels of adiponectin, leptin, resistin, and TNF-*α* were measured using the enzyme-linked immunosorbent assay (ELISA) method. *Results*. Significant lower levels of adiponectin were present (*P* = 0.0061) in PAOD patients (2380.23 ± 1634.42 pg/mL) compared to the control group (3065.06 ± 1901.2 pg/mL). The mean value of leptin (2844.42 ± 3301.08 pg/mL) and resistin (2047.81±3301.08 pg/mL) patients included in the PAOD group was higher, as compared to the control group. Statistically significant difference was found between the two groups for leptin (*P* = 0.0332) and for resistin (*P* = 0.0352). No statistically significant difference for TNF-*α* was found between the two groups (*P* > 0.05). *Conclusion*. The markers of inflammation secreted by the adipose tissue (adiponectin, leptin, resistin) showed significant differences in patients from the case group (with PAOD) compared to the control group.

## 1. Introduction

Atherosclerosis is the primary cause of coronary heart disease (CHD) and of peripheral arterial occlusive disease (PAOD), being one of the most common causes for disease and death throughout the world [1]. It has been found that the adipose tissue is connected with the pathogenesis of atherosclerosis as a result of a secretion of a multitude of pro- and antiatherogenic cytokines and adipokines such as adiponectin, leptin, resistin, and acute-phase proteins [2]. These adipokines play a central role in the pathogeny of the metabolic syndrome [3, 4] and in the development of systemic inflammation [5, 6].

The serum levels of adiponectin, potential mediator of atherosclerosis, are significantly low in the case of patients with visceral obesity and insulin-resistance as well as in the case of type 2 diabetes mellitus [4, 6, 7]. The low levels of adiponectin were associated with the presence of CHD [6, 8] and appear to be a risk factor for CHD [6, 9].

Besides adiponectin, the adipokines intensely produced by the adipose tissue are leptin and resistin. The physiological role of leptin is very complex; it could be an important mediator of the relationship among obesity, overweight, and atherosclerosis [3, 5]. Resistin, also involved in endothelial dysfunction, expressed primarily in inflammatory cells, suggests a potential role in atherosclerosis [10, 11]. However, the relationship between resistin and inflammation, insulin resistance, and atherosclerosis in humans remains unexplored. Tumor necrosis factor-*α* (TNF-*α*), a pleiotropic proinflammatory cytokine, has been also recognized in human atheroma [12].

Adiponectin, leptin, and resistin are adipocytokines that control body weight, and insulin sensitivity [13]. These adipokines have been investigated in relation to obesity, metabolic syndrome, insulin resistance, and other pathological conditions and processes. In addition, it is now established that adipokines play a role in the maintenance of an inflammatory state in adipose tissue and in the development of obesity and comorbidities [14].

It has become increasingly evident that white adipose tissue-derived cytokines mediate between obesity-related exogenous factors and the molecular events that lead to metabolic syndrome, inflammation, and cardiovascular diseases [15]. Although there are studies which demonstrate the relationship between adipokines, obesity, and increased risk of cardiovascular events [14–16], their interplay is still poorly studied.

The hypothesis of the study starts from the assumption that among people aged over 55 atherosclerotic PAOD is a known indicator of systemic atherosclerotic disease [17] and literature in this area is scarce [18–20]. The role of adiponectin in the initiation and development of atherosclerotic lesions in PAOD patients has been studied and analyzed very little so far [21]. At the same time remains unexplored the role of leptin and resistin in PAOD. In the absence of data on relationship between adipokines and atherosclerotic PAOD, we have decided to undertake the present study.

The aim of our study was to assess the serum levels of adipokines (adiponectin, leptin, resistin, and TNF-*α*) in patients with PAOD caused by atherosclerosis. Furthermore, the link between serum levels within investigated adipokines on one hand and between adipokines and cholesterol, HDL cholesterol, triglycerides, glycemia, fibrinogen, or CRP (C reactive protein), smoking, obesity, arterial hypertension, type II diabetes mellitus, coronary heart disease on the other hand was investigated.

## 2. Materials and Methods

### 2.1. Study Protocol

A prospective case-control study was carried out at the Surgical Clinic No. 2 of the County Clinical Hospital of Cluj-Napoca, Romania, from January 2009 to January 2011.

The case group included patients with PAOD caused by atherosclerosis, diagnosed by duplex scanning, based on an ankle-brachial index (ABI) < 0.9. The ankle-brachial index was calculated by dividing the ankle systolic pressure measured at the malleolar level with a blood pressure cuff, to the higher of the two brachial pressures.

Patients with terminal diseases (e.g., cardiovascular failure, hepatic or renal failure, etc.) were excluded from the case group. Only the patients who agreed to participate in the study were included.

The control group included matched patients from our outpatients referred to us for lower limb chronic venous insufficiency problems, general surgery conditions (e.g., appendicitis, hernia, cholecystitis, etc.), or hospital staff and their relatives that presented an ABI > 0.9. The subjects included in this group had no known history of autoimmune diseases, neoplasia, cardiovascular failure, and hepatic or renal failure.

The study was approved by the Local Ethics Committee (both University's and Hospital's) and was conducted according to the Helsinki Declaration of 1964 as revised in 1983. Each subject included in the study (both case and control group) signed a written informed consent for their participation in the study. All subjects participated voluntary and the confidentiality of all data was respected.

The following data were collected for both groups: measurements of basic constituents (age, weight, height, and distal arterial pressures), medical history (arterial hypertension (AHT), type II diabetes mellitus (DM), coronary heart disease (CHD)), and drug use (e.g., actual or former smoking habit—more than 10 cigarettes/day and alcohol abuse). From each subject included in the study, we obtained a blood sample and determined the following parameters: cholesterol, triglycerides, fibrinogen, high-density lipoprotein (HDL), glycemia, creatinine, adiponectin, leptin, resistin, TNF-*α*, and C reactive protein (CRP). To all subjects included in the study the blood samples were collected after an overnight fast, in the morning between 7 and 9 AM. Plasma concentrations of glucose, creatinine, lipids, and lipoproteins were determined using standard enzymatic colorimetric methods. Normal serum concentration of CRP was considered normal at values lower than 1 mg/dL. Total cholesterol > 200 mg/dL was used as a cutoff value for the lipid profile according to Adult Treatment Panel III guidelines [22].

Circulating plasma levels of adiponectin, leptin, resistin and TNF-*α* were measured by a commercially available enzyme-linked immunosorbent assay (ELISA), using Quantikine reagents (R&D Systems): Human Adiponectin, Human Leptin, Human Resistin, and Human TNF-*α*/TNFSF1A.

Body mass index (BMI) was calculated according to Quetelet's formula: weight (kg)/height (m^2^) [23]. The following classification of subjects according to BMI was applied to both case and control group: BMI < 25 kg/m^2^ = normal, 25 ≤ BMI < 30 kg/m^2^ = overweight, and BMI ≥ 30 kg/m^2^ = obese (over 40, morbidly obese). Patients were considered as hypertensive according to Joint British Societies' guidelines, patients with systolic blood pressure higher than 140 mmHg or with diastolic blood pressure higher than 90 mmHg, or both [24]. Coronary heart disease (CHD) was defined by standard Framingham Heart Study criteria as any of new-onset angina, coronary insufficiency, or fatal or nonfatal myocardial infarction [25]. Diabetes mellitus (DM) was defined according to the American Diabetes Association as follows: any patient with two fasting plasma glucose levels of 126 mg/dL (7.0 mmol/L) or higher, or treatment with blood glucose-lowering drugs [26].

### 2.2. Statistical Analysis

Descriptive statistics for quantitative data were expressed as mean, standard deviation, and 95% confidence intervals whenever the data were normal distributed. Otherwise, the median was used. The comparisons between groups for quantitative data were carried out with Student's *t*-test when data were normally distributed; otherwise the Mann-Withney test was applied.

Qualitative variables were summarized as absolute frequency, percentages and associated 95% confidence intervals. The 95% confidence interval was calculated based on optimized binomial distribution formula [27, 28]. Comparison between two proportions was performed by applying the *Z*-test. Statistical analysis was carried out with SPSS v. 16; all tests were applied at a significance level of 5%.

The link between the investigated adipokines and cholesterol, HDL cholesterol, triglycerides, glycemia, fibrinogen, or CRP was investigated using Pearson correlation coefficient if experimental data proved to be normally distributed; otherwise Spearman correlation coefficient was used. The link between the investigated adipokines and the qualitative variables (such as obesity, AHT (yes/no), DM (yes/no), CHD (yes/no), and smoking (yes/no)) was carried out by applying the Spearman's rank correlation coefficient. The power of the correlation was interpreted according to Colton rules [29]. A correlation coefficient was considered statistically significant when the *P* value was smaller than 0.05.

## 3. Results

### 3.1. Studied Groups

One hundred and forty patients, 26 females (18.6%, 95%CI (12%–26%)) and 114 males (81.4%, 95%CI (74%–88%)), met the inclusion criteria and were studied in the case group. Eighty-one patients, 17 females (21%, 95%CI (12%–31%)) and 64 males (79%, 95%CI (69%–88%)), were included in the control group. No statistically significant difference was identified between the proportion of females in both case and control group (*P* = 0.6647).

The mean age in the case group was of 65.25 ± 10.84 years (95%CI (63.44–67.06)), with a median of 65.50. A statistically significant age difference was identified between the female patients (70.35 ± 10.33, 95%CI (66.18–74.52), median = 72) and the male patients (64.09 ± 10.66, 95%CI (62.11–66.07)) in the case group (Mann-Withney statistics = 989, *P* = 0.0082).

The mean age of control group was of 60.83 ± 10.60 years (95% CI (58.48–63.17)), with a median of 60. No statistically significant age difference was identified between the female population (65.06 ± 10.87, 95%CI (59.47–70.650), median = 68) and the male population (59.70 ± 10.33, 95%CI (57.12–62.28)) in the control group (Mann-Whitney statistics = 412, *P* = 0.1258).

A statistically significant difference regarding the age of the subjects in the case and control group was identified (Mann-Whitney statistics = 4320, *P* = 0.0032).

The mean of BMI of case group was of 26.54 ± 4.58 (*n* = 140); the mean of BMI of control group was of 26.23 ± 4.64. No statistically significance difference regarding the BMI between the case and the control group was identified (Student's *t*-test = 0.483, df (degrees of freedom) = 219, *P* value = 0.629).

### 3.2. Case versus Control Comparison

The comparison between case and control featuring number of patients, percentages, and associated *Z*-scores considering associated diseases is presented in Table 1.

The biochemical characteristics of both case and control group are presented in Table 2. 

The serum levels of the inflammation parameters synthesized by the adipose tissue determined by the described immunological techniques are presented in Table 3. 

Adiponectin presented statistically significant (*P* = 0.0061, Table 3) lower levels in PAOD patients (2380.23 ± 1634.42 pg/mL) than in control group patients (3065.06 ± 1901.2 pg/mL). The serum levels of leptin, resistin, and CRP proved to be also statistically significant higher in the case group compared to the control group (*P* < 0.05, Table 3). 

The serum levels of leptin and resistin were also monitored, levels which had higher mean values in PAOD patients compared to control group patients, hence the statistically significant differences (Table 3). 

TNF-*α* did not present significant differences between the two investigated groups (Table 3). 

**Figure 1:**
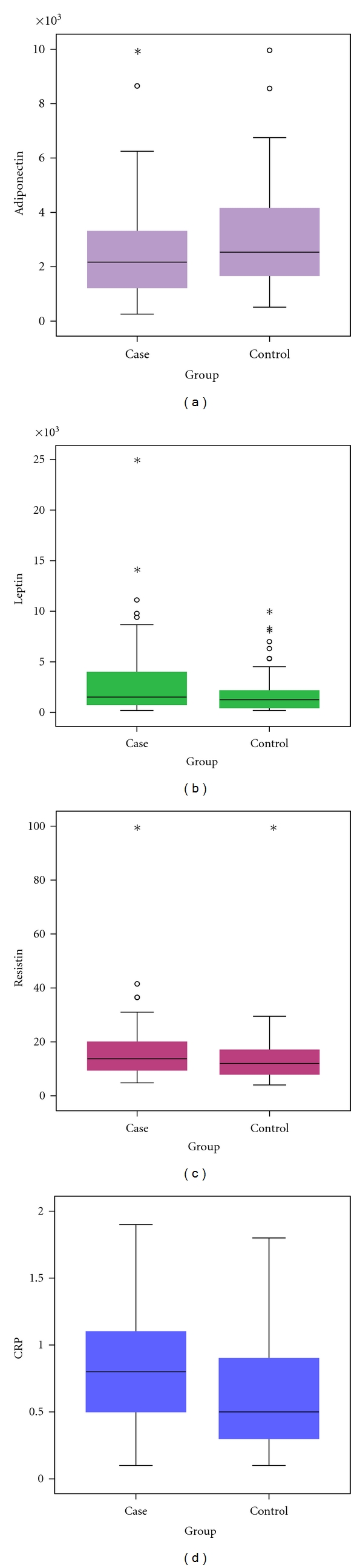
The graphic representation of the serum levels ranges of the inflammation markers (adipokines, CRP) in the PAOD patients and the control group.

The graphical representation of the ranges associated to the markers of the inflammation secreted by the adipose tissue is presented in Figures 1(a)–1(d). 

Eighty patients (24 from case group and 65 from control group) had the TNF-*α* over the normal value. Out of these patients, almost 73% were normal weighted or overweighed, 27% were obese. 

The correlation analysis carried out on adipokines identified the following statistically significant correlations; the results are presented in Table 4. 

All other correlations within adipokines and between adipokines and all investigated variables did not prove to be statistically significant (*P* > 0.05).

## 4. Discussion 

The levels of adipokines (adiponectin, leptin, resistin, and TNF-*α*) in patients with PAOD caused by atherosclerosis were successfully investigated. The group of PAOD patients was compared to a control group. The sampling of the patients in both case and control group was as accurate as possible. Unfortunately, statistically significant differences were identified for the following variables: age, AHT, and CHD. The age of the patients in case and control group proved to be a statistically significant difference, but this variable did not affect the results of our study since in the medical specialty literature no statistically significant difference in adipokines level was reported [30]. No statistically significant difference was identified between the two studied groups for BMI. The presence of a statistically significant difference between groups in terms of BMI could be considered a bias factor since the medical specialty literature proved an inverse correlation between adiponectin and fat mass [31]. 

The absence of a statistically significant difference in the proportion of genders between groups proves the proper assignment of patients in the groups. A higher statistically significant proportion of patients from our case group proved to have had AHT compared to control group (*P* < 0.05, see Table 1) [32]. Moreover, a higher statistically significant proportion of patients from the case group proved to have had CHD compared to the control group (*P* < 0.05, see Table 1) [11, 32–34]. 

The absence of a statistically significant difference between the percentages of diabetes mellitus in both case and control groups confirms the reliability of our results, knowing that this disease is an important risk factor for large vessel atherosclerotic occlusive disease [10, 32]. Furthermore, even if smoking was identified as being closely linked to PAOD, a relation first identified by Erb in 1911 [34–36], the distribution of smoking in our group's patients did not prove to be statistically significant different (*P* = 0.0545, see Table 1). The groups under investigation were homogeneous as far as the demographic data and clinical characteristics were concerned, except for the age variable and the associated cardiovascular diseases. 

Hyperlipidemia has been associated with an increased rate of lower extremity occlusive disease (*P* = 0.049, Table 2), and our results were according to the data published by the medical specialty literature [37]. Even if some studies identified the cholesterol serum level as an important independent risk factor, in our study it was not associated with PAOD [36]. 

No statistically significant differences could be identified for cholesterol, HDL, Creatinine, and glucose serum levels (Table 2). 

The fibrinogen proved to be statistically different in our study when the two investigated groups were compared (higher values in the case group compared to control group, *P* < 0.05, see Table 2). 

Another acute phase protein, CRP, which normally increases not only in infections but also in activations of certain chronic diseases, was also significantly higher in the investigated PAOD patients (*P* < 0.05, Table 3). 

In our study the serum values of adiponectin were significantly lower in the case group than the values of the control group (Table 3). This result could indicate that hypoadiponectinemia is related to the development of PAOD. Since that low levels of adiponectin are correlated to endothelial dysfunction [38], the results of our study suggest that hypoadiponectinemia can be used as a marker of atherosclerosis. Because almost a third of patients included in the study were obese or diabetic, further studies on larger sample size must be carried out for the association between adipokines and CRP and PAOD in the nonobese/diabetic population. 

With regard to the role of leptin in the development of atherosclerotic lesions in PAOD, our study has indicated significantly increased serum levels in these patients compared to control group (*P* < 0.05, Table 3). Knowing its proatherogenic function, correlated with the increased values that we had detected, recommends this parameter as a possible biomarker of atherosclerosis with peripheral vascular localizations [39–41]. 

The mean values of resistin presented significantly higher values in PAOD patients compared to the patients in the control group, and this fact suggested its possible participation in the process of atherogenesis (*P* < 0.05, Table 3). As far as we know there are no published data available to appreciate the role of resistin in the case of PAOD; therefore more studies are required. 

A series of positive or negative significant correlations were identified between certain adipokines and some of the investigated parameters (see Table 4) but all these correlations proved to be weak. Leptin proved to be significantly related with triglyceride serum level, positively for case group and negatively for control group. Adiponectin proved to be significantly related with AHT, positively for case group and negatively for control group. In our study, adipokine levels did not prove to be related to obesity or to BMI, a fact contradicted by some studies [31]. Adiponectin and leptin proved to be significantly related to TNF-*α* in control group and obesity was also related to TNF-*α* (Table 4), confirming data from the literature [7, 8, 42]. 

The weak negative relationship (*P* < 0.05, Table 4) between TNF-*α* and adiponectin confirmed the already known antagonistic effect of the adiponectin on the TNF-*α* activity [43–46].

**Study Strengths and Limitations**

Adipokines expression has been extensively studied in cardiovascular disease but all studies have been performed either on patients with typical ischaemic heart disease or in hypertensive patients [10–12, 15]. However, except for a recent report by Dieplinger et al. [21], there had been no other data on adipokines expression in PAOD. More important, Dieplinger et al. [21] studied only adiponectin expression in PAOD and the results are similar to our findings. This is a pilot study designed to assess if inflammatory and metabolic mediators can be used as potential atherosclerotic biomarkers in PAOD patients. Here, we have showed that serum adiponectin, resistin, and leptin might have a potential role in developing atherosclerotic lesions in patients with PAOD. Further experimental and clinical studies are required to establish the exact role of adipokines in the disease development and to provide a definite answer to the question of whether measuring these chemokines could be used for the follow-up of PAOD patients.

## 5. Conclusion 

The markers of the inflammation secreted by the adipose tissue (adiponectin, leptin, resistin, CRP) showed significant differences in case group patients (with PAOD) compared to control group. Our results point out the possible role of hypoadiponectinemia in developing PAOD. Proinflammatory cytokines produced by white adipose tissue (resistin and leptin) are likely to be promising targets for controlling the reduction of inflammation in order to prevent the progression of early or already established atherosclerotic lesions in PAOD patients, but further research is, however, necessary. 

## Figures and Tables

**Table 1:** Associated diseases: comparison between case and control group.

	Group		*Z*-test to compare	
			proportions	
	Case^a^ *n* (%)	Control^b^ *n* (%)	*Z*	*P* value
Obesity	33 (23.57)	24 (29.63)	−0.9613	0.3364
DM	45 (32.14)	27 (33.33)	−0.1528	0.8786
AHT	95 (67.86)	39 (48.15)	2.9374	0.0033
CHD	106 (75.71)	31 (38.27)	5.8555	<0.0001
Smoking status	97 (69.29)	45 (55.56)	1.9231	0.0545

^a^: sample size case group = 140 patients; ^b^: sample size control group = 81 subjects; DM: Diabetes mellitus; AHT: arterial hypertension; CHD: coronary heart disease.

**Table 2:** Biochemical data for PAOD patients and controls.

		Group		Mann-Whitney test	
		Case	Control	Statistics	*P* value
Cholesterol (mg/dL)	Mean ± StDev	201.54 ± 49.23	189.48 ± 55.12	4869	0.0803
	Median	193.00	190.00		

TG (mg/dL)	Mean ± StDev	130.62 ± 56.97	116.81 ± 52.01	4769	0.0490
	Median	117.00	105.00		

HDL (mg/dL)	Mean ± StDev	48.23 ± 13.01	46.65 ± 11.57	5295	0.4120
	Median	48.00	46.00		

Creatinine (mg/dL)	Mean ± StDev	0.89 ± 0.21	0.84 ± 0.21	4829	0.0614
	Median	0.90	0.90		

Glycemia (mg/dL)	Mean	99.97 ± 22.63	98.32 ± 18.04	5494	0.7006
	Median	94.00	95.00		

Fibrinogen (mg/dL)	Mean	341.66 ± 84.07	318.68 ± 72.20	4725	0.0390
	Median	338.00	311.00		

TG: triglycerides; HDL: high-density lipoprotein; StDev: Standard deviation.

**Table 3:** Serum concentrations of the studied parameters in the different studied groups.

		Group		Mann-Whitney test	
		Case	Control	Statistics	*P* value
Adiponectin (pg/mL)	Mean ± StDev	2380.23 ± 1634.42	3065.06 ± 1901.2	4415	0.0061
	Median	2170.00	2536.00		

Leptin (pg/mL)	Mean ± StDev	2844.42 ± 3301.08	2047.81 ± 3301.08	4695	0.0332
	Median	1515.00	1254.00		

Resistin (pg/mL)	Mean ± StDev	16.14 ± 10.68	13.84 ± 11.20	4706	0.0352
	Median	13.75	12.00		

TNF-*α* (pg/mL)	Mean ± StDev	4.96 ± 5.18	5.11 ± 6.01	5540	0.7756
	Median	3.30	2.90		

CRP (mg/dL)	Mean ± StDev	0.82 ± 0.45	0.62 ± 0.41	4131	0.0008
	Median	0.80	0.50		

TNF-*α*: tumor necrosis factor-*α*; CRP: C reactive protein; StDev: standard deviation.

**Table 4:** The results of correlation analysis.

Variables	Correlation		
	Direction	Power	*P* value
Case group			
TNF-*α* versus DM	Positive	Weak	0.0341
Resistin versus AHT	Negative	Weak	0.0080
Adiponectin versus AHT	Positive	Weak	0.0313
Leptin versus cholesterol	Positive	Weak	0.0437
Leptin versus triglycerides	Positive	Weak	0.0090
Control group			
TNF-*α* versus obesity	Negative	Week	0.0468
TNF-*α* versus adiponectin	Negative	Weak	0.0222
TNF-*α* versus leptin	Positive	Weak	0.0255
Leptin versus triglycerides	Negative	Weak	0.0238
Adiponectin versus AHT	Negative	Weak	0.0359
Obesity versus HDL cholesterol	Positive	Week	0.0342

Week correlation was considered whenever the correlation coefficient was between [−0.5; 0.5].

## References

[1] The World Health Organization (WHO) Reducing Risks, Promoting Healthy Life. http://www.who.int/whr/2002/en.

[2] Calabrò P, Golia E, Maddaloni V (2009). Adipose tissue-mediated inflammation: the missing link between obesity and cardiovascular disease?. *Internal and Emergency Medicine*.

[3] Maury E, Brichard SM (2010). Adipokine dysregulation, adipose tissue inflammation and metabolic syndrome. *Molecular and Cellular Endocrinology*.

[4] Mathieu P, Pibarot P, Després JP (2006). Metabolic syndrome: the danger signal in atherosclerosis. *Vascular Health and Risk Management*.

[5] Guzik TJ, Mangalat D, Korbut R (2006). Adipocytokines—novel link between inflammation and vascular function?. *Journal of Physiology and Pharmacology*.

[6] Berg AH, Scherer PE (2005). Adipose tissue, inflammation, and cardiovascular disease. *Circulation Research*.

[7] Koenig W, Khuseyinova N, Baumert J, Meisinger C, Löwel H (2006). Serum concentrations of adiponectin and risk of type 2 diabetes mellitus and coronary heart disease in apparently healthy middle-aged men. Results from the 18-year follow-up of a large cohort from Southern Germany. *Journal of the American College of Cardiology*.

[8] Broedl UC, Lebherz C, Lehrke M (2009). Low adiponectin levels are an independent predictor of mixed and non-calcified coronary atherosclerotic plaques. *PLoS ONE*.

[9] Kumada M, Kihara S, Sumitsuji S (2003). Association of hypoadiponectinemia with coronary artery disease in men. *Arteriosclerosis, Thrombosis, and Vascular Biology*.

[10] Reilly MP, Lehrke M, Wolfe ML, Rohatgi A, Lazar MA, Rader DJ (2005). Resistin is an inflammatory marker of atherosclerosis in humans. *Circulation*.

[11] Kawanami D, Maemura K, Takeda N (2004). Direct reciprocal effects of resistin and adiponectin on vascular endothelial cells: a new insight into adipocytokine-endothelial cell interactions. *Biochemical and Biophysical Research Communications*.

[12] Anfossi G, Russo I, Doronzo G, Pomero A, Trovati M (2010). Adipocytokines in atherothrombosis: focus on platelets and vascular smooth muscle cells. *Mediators of Inflammation*.

[13] Delporte M-L, El Mkadem SA, Quisquater M, Brichard SM (2004). Leptin treatment markedly increased plasma adiponectin but barely decreased plasma resistin of ob/ob mice. *American Journal of Physiology*.

[14] Lago F, Gómez R, Gómez-Reino JJ, Dieguez C, Gualillo O (2009). Adipokines as novel modulators of lipid metabolism. *Trends in Biochemical Sciences*.

[15] Gualillo O, González-Juanatey JR, Lago F (2007). The emerging role of adipokines as mediators of cardiovascular function: physiologic and clinical perspectives. *Trends in Cardiovascular Medicine*.

[16] Windham BG, Griswold ME, Farasat SM (2010). Influence of leptin, adiponectin, and resistin on the association between abdominal adiposity and arterial stiffness. *American Journal of Hypertension*.

[17] Hooi JD, Kester ADM, Stoffers HEJH, Rinkens PELM, Knottnerus JA, Van Ree JW (2004). Asymptomatic peripheral arterial occlusive disease predicted cardiovascular morbidity and mortality in a 7-year follow-up study. *Journal of Clinical Epidemiology*.

[18] Murabito JM, Keyes MJ, Guo CY (2009). Cross-sectional relations of multiple inflammatory biomarkers to peripheral arterial disease: the Framingham offspring study. *Atherosclerosis*.

[19] Wild SH, Byrne CD, Tzoulaki I (2009). Metabolic syndrome, haemostatic and inflammatory markers, cerebrovascular and peripheral arterial disease: the Edinburgh artery study. *Atherosclerosis*.

[20] Dieplinger B, Poelz W, Haltmayer M, Mueller T (2007). Association of adiponectin and amino terminal proBNP in peripheral arterial disease. *Clinica Chimica Acta*.

[21] Dieplinger B, Poelz W, Haltmayer M, Mueller T (2006). Hypoadiponectinemia is associated with symptomatic atherosclerotic peripheral arterial disease. *Clinical Chemistry and Laboratory Medicine*.

[22] (2002). Third report of the national cholesterol education program (NCEP) expert panel on detection, evaluation, and treatment of high blood cholesterol in adults (Adult Treatment Panel III) final report. *Circulation*.

[23] Eknoyan G (2008). Adolphe Quetelet (1796–1874)—the average man and indices of obesity. *Nephrology Dialysis Transplantation*.

[24] Wood D, Wray R, Poulter N (2005). JBS 2: Joint British Societies' guidelines on prevention of cardiovascular disease in clinical practice. *Heart*.

[25] Frankel DS, Vasan RS, D’Agostino RB (2009). Resistin, adiponectin, and risk of heart failure. The Framingham offspring study. *Journal of the American College of Cardiology*.

[26] Kahn R (1997). Report of the Expert Committee on the Diagnosis and Classification of Diabetes Mellitus. *Diabetes Care*.

[27] Jäntschi L, Bolboacǎ SD (2010). Exact probabilities and confidence limits for binomial samples: applied to the difference between two proportions. *TheScientificWorldJOURNAL*.

[28] Bolboacă SD, Jäntschi L (2008). Optimized confidence intervals for binomial distributed samples. *International Journal of Pure and Applied Mathematics*.

[29] Colton T (1974). *Statistics in Medicine*.

[30] Stenholm S, Metter EJ, Roth GS (2011). Relationship between plasma ghrelin, insulin, leptin, interleukin 6, adiponectin, testosterone and longevity in the Baltimore Longitudinal Study of Aging. *Aging*.

[31] Marra F, Bertolani C (2009). Adipokines in liver diseases. *Hepatology*.

[32] Ouriel K (2001). Peripheral arterial disease. *Lancet*.

[33] Krȩcki R, Drozdz J, Szcześniak P, Orszulak-Michalak D, Krzemińska-Pakuła M (2008). Novel atherogenesis markers for identification of patients with a multivessel coronary artery disease. *Kardiologia Polska*.

[34] Kopff B, Jegier A (2005). Adipokines: adiponectin, leptin, resistin and coronary heart disease risk. *Przegląd lekarski*.

[35] Erb W (1911). Klinische beiträge zur pathologie des intermittieren den hinkens. *Münchener Medizinische Wochenschrift*.

[36] Smith I, Franks PJ, Greenhalgh RM, Poulter NR, Powell JT (1996). The influence of smoking cessation and hypertriglyceridaemia on the progression of peripheral arterial disease and the onset of critical ischaemia. *European Journal of Vascular and Endovascular Surgery*.

[37] Zimmerman BR, Palumbo PJ (1981). A prospective study of peripheral occlusive arterial disease in diabetes—III. Initial lipid and lipoprotein findings. *Mayo Clinic Proceedings*.

[38] Tan KCB, Xu A, Chow WS (2004). Hypoadiponectinemia is associated with impaired endothelium-dependent vasodilation. *Journal of Clinical Endocrinology and Metabolism*.

[39] Hou N, Luo J-D (2011). Leptin and cardiovascular diseases. *Clinical and Experimental Pharmacology and Physiology*.

[40] Elbatarny HS, Netherton SJ, Ovens JD, Ferguson AV, Maurice DH (2007). Adiponectin, ghrelin, and leptin differentially influence human platelet and human vascular endothelial cell functions: implication in obesity-associated cardiovascular diseases. *European Journal of Pharmacology*.

[41] Napoleone E, Di Santo A, Amore C (2007). Leptin induces tissue factor expression in human peripheral blood mononuclear cells: a possible link between obesity and cardiovascular risk?. *Journal of Thrombosis and Haemostasis*.

[42] Recasens M, Ricart W, Fernández-Real JM (2004). Obesity and inflammation. *Revista de medicina de la Universidad de Navarra*.

[43] Hotamisligil GS (1999). Mechanisms of TNF-*α*-induced insulin resistance. *Experimental and Clinical Endocrinology and Diabetes*.

[44] Fasshauer M, Klein J, Neumann S, Eszlinger M, Paschke R (2001). Tumor necrosis factor *α* is a negative regulator of resistin gene expression and secretion in 3T3-L1 adipocytes. *Biochemical and Biophysical Research Communications*.

[45] Ouchi N, Kihara S, Arita Y (2000). Adiponectin, an adipocyte-derived plasma protein, inhibits endothelial NF-*κ*B signaling through a cAMP-dependent pathway. *Circulation*.

[46] Yamauchi T, Kamon J, Waki H (2003). Globular adiponectin protected ob/ob mice from diabetes and ApoE-deficient mice from atherosclerosis. *Journal of Biological Chemistry*.

